# Cytochrome P450 Family 1 Inhibitors and Structure-Activity Relationships

**DOI:** 10.3390/molecules181214470

**Published:** 2013-11-25

**Authors:** Jiawang Liu, Jayalakshmi Sridhar, Maryam Foroozesh

**Affiliations:** Department of Chemistry, Xavier University of Louisiana, New Orleans, LA 70125, USA

**Keywords:** cytochrome P450, enzyme inhibitor, stilbenoids, flavonoids, structure activity relationships, mechanism-based inhibitor

## Abstract

With the widespread use of *O*-alkoxyresorufin dealkylation assays since the 1990s, thousands of inhibitors of cytochrome P450 family 1 enzymes (P450s 1A1, 1A2, and 1B1) have been identified and studied. Generally, planar polycyclic molecules such as polycyclic aromatic hydrocarbons, stilbenoids, and flavonoids are considered to potentially be effective inhibitors of these enzymes, however, the details of the structure-activity relationships and selectivity of these inhibitors are still ambiguous. In this review, we thoroughly discuss the selectivity of many representative P450 family 1 inhibitors reported in the past 20 years through a meta-analysis.

## 1. Introduction

Cytochrome P450 enzymes are a large ubiquitous superfamily of enzymes, playing a significant physiological role in the detoxification of xenobiotics, and the biosynthesis of many endogenous compounds. P450 families 1, 2, and 3 contribute most extensively to the biotransformation of xenobiotics into more polar metabolites that are readily excreted. In humans and most mammals, P450 family 1 comprises three well-studied monooxygenases, 1A1, 1A2, and 1B1.

Cytochrome P450 1A1, a well-known aryl hydrocarbon hydroxylase, is implicated in the metabolic activation of environmental procarcinogens such as polycyclic aromatic hydrocarbons (PAHs) and polyhalogenated aromatic hydrocarbons (PHAHs). Human P450 1A1 is mainly expressed in extrahepatic tissues such as lung, gastrointestinal tract, placenta, and skin, and is present only at low levels in the liver [1,2]. P450 1A1 is one of the most important enzymes involved in tumorigenesis initiated by environmental pollutants [3]. A number of epidemiological studies have shown that genetic variants of human *CYP1A1* gene are significantly associated with the susceptibilities to lung and breast cancers [4,5,6]. Because of the significant role of P450 1A1 enzyme in human carcinogenesis, modulation of P450 1A1 activity has been considered as a potential target for cancer chemoprevention.

P450 1A2 is one of the major cytochrome P450 enzymes in human liver (about 13%) responsible for the metabolism of a variety of arylamines and heterocyclic arylamines which include numerous therapeutic drugs such as phenacetin, lidocaine, tacrine, and theophylline [2,7]. P450 1A2 is notable for the capacity to *N*-oxidize arylamines, bioactivating arylamines such as 2-amino-3-methyl-imidazo[4,5-f]quinoline (IQ) and 2-amino-1-methyl-6-phenylimidazo[4,5-b]pyridine (PhIP) into potent mutagenic or carcinogenic compounds [8]. Procarcinogen activation by P450 1A2 has a significant effect on an individual’s susceptibility to cancer [8]. Inhibition of P450 1A2 also has implications in cancer prevention.

Cytochrome P450 1B1 is found mainly in extrahepatic tissues as well as in a variety of human tumors. This has important implications for tumor development and progression. Metabolic activation of 17β-estradiol (E2) to 4-hydroxy-17β-estradiol by P450 1B1 has been postulated to be an important factor in mammary carcinogenesis [9,10,11]. Like P450 1A1, this enzyme also metabolically activates procarcinogens to carcinogenic forms, resulting in mutagenesis and tumorigenesis [9]. Thus, regulators of the expression and catalytic activity of P450 1B1 have been suggested to play an important role in cancer chemoprevention, especially at the early stage of tumorigenesis [12]. Recently, it has been observed that P450 1B1 could generate reactive oxygen species resulting in hypertension, which suggested that P450 1B1 inhibitors might be clinically useful in the treatment of hypertension and associated cardiovascular diseases [13].

To summarize, although P450 family 1 cytochromes are responsible for the metabolism of a wide variety of xenobiotics as well as endogenous compounds, the enzymatic reactions also produce reactive intermediates which can covalently bond to cellular macromolecules leading to toxicity, mutagenicity, and carcinogenicity. Hence, much emphasis has been on the inhibition of these enzymes to develop potential cancer preventative and therapeutic agents [2,3,9,14]. The potential anti-cancer strategies targeting P450 inhibition were postulated as: (i) preventing the conversion of environmental procarcinogens to active carcinogens; (ii) preventing the conversion of hormonal precursors to carcinogenic hormone derivatives; and (iii) preventing the metabolic inactivation of anti-cancer drugs [15]. Herein we will review multifarious inhibitors of cytochrome P450 family 1 enzymes, 1A1, 1A2, and 1B1.

## 2. Classified Inhibitors of P450s 1A1, 1A2, and 1B1

Because of their high structural and functional similarity, cytochrome P450s 1A1, 1A2, and 1B1 are known to have overlapping substrates, inducers, and inhibitors. Therefore, the development of selective inhibitors towards any of these enzymes is a great challenge. To date, thousands of compounds (including natural products, pharmaceuticals, and synthetic compounds) have been evaluated for their inhibitory activities toward P450 family 1 enzymes. In an early study, the inhibition effects of 12 compounds toward P450s 1A1 and 1A2 were investigated, including α-naphthoflavone, ellipticine, 7-ethoxyresorufin, 5-methoxypsoralen, 8-methoxypsoralen, 7-ethoxycoumarin, nifedipine, propranolol, caffeine, paraxanthine, theophylline, and theobromine [16]. The comparison between groups indicated that polycyclic aromatic hydrocarbons, flavones, and coumarins were highly potent P450 inhibitors with IC_50_ values in the micromolar level. This review mainly focuses on the highly potent inhibitor groups, which are more likely to become chemopreventive agents.

### 2.1. Polycyclic Aromatic Hydrocarbons

**Figure 1:**
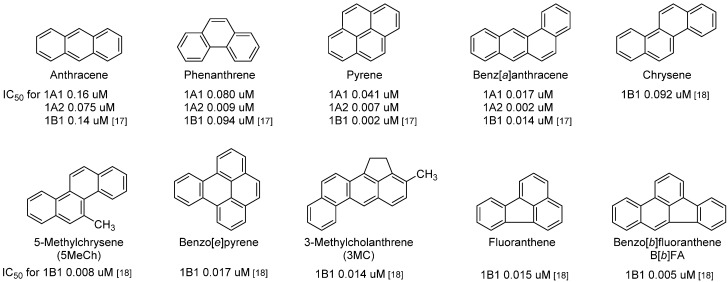
Polycyclic aromatic hydrocarbons as P450 family 1 inhibitors.

Since PAHs such as benzo[a]pyrene (B[a]P) are classical substrates of P450 family 1 enzymes, a number of PAHs were examined as inhibitors of the three enzymes through 7-ethoxyresorufin *O*-deethylation (EROD) assay by Shimada *et al*. The simplest PAHs, anthracene and phenanthrene (Figure 1), showed stronger inhibition toward P450 1A2 over the other two enzymes. On the other hand, the four-ring pyrene structure showed extremely strong inhibition of P450 1B1 with an IC_50_ of 2 nM, but not much selectivity relative to P450 1A2 [17]. The most potent inhibitor of P450 1A2 was benz[a]anthracene (IC_50_ = 2 nM), exhibiting 8.9-fold and 7.0-fold more effective inhibition of this enzyme relative to P450s 1A1 and 1B1, respectively [17]. A follow-up study showed that the complex PAHs (Figure 1), such as chrysene, benzo[e]pyrene, 3-methylcholanthrene (3MC), fluoranthene (FA), and benzo[*b*]fluoranthene (B[b]FA), exhibited nanomolar level inhibition of P450 1B1-mediated EROD activity [18]. Mechanism investigation showed that all the studied non-substituted PAHs acted as competitive inhibitors [17,18].

Polycyclic aromatic hydrocarbons appear to be strong inhibitors of P450s 1A1, 1A2, and 1B1. However, PAHs (such as benzo[a]pyrene, 3-methylcholanthrene, and 7,12-dimethylbenz[a]anthracene (DMBA)) have also been linked to tumor formation and cancer in humans. Thus, even though PAHs have already become useful tools to investigate the structure and function of P450 family 1 enzymes, it is not logical to design therapeutic agents using them.

### 2.2. Naphthoquinones and Anthraquinones

**Figure 2:**
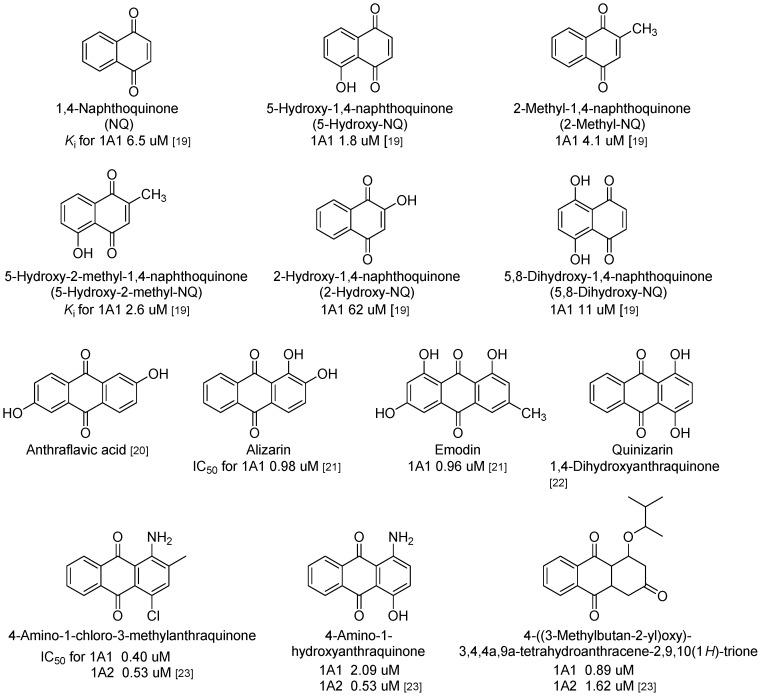
Structures of naphthoquinone and anthraquinone derivatives.

Naphthoquinones and anthraquinones could be considered as oxygen-substituted naphthalenes and anthracenes, respectively. Various naphthoquinone derivatives, including 1,2-naphthoquinone and 1,4-naphthoquinone, are known ingredients of Chinese traditional medicines. The inhibitory effects of six derivatives of 1,4-naphthoquinone (NQ) on rat cytochrome P450 1A1-dependent EROD activity were examined using yeast microsomes containing overexpressed rat P450 1A1. Of these NQs, 5-hydroxy-2-methyl-NQ, 2-methyl-NQ, 2-hydroxy-NQ, and NQ showed competitive inhibition, whereas 5,8-dihydroxy-NQ and 5-hydroxy-NQ showed non-competitive inhibition [19]. The *K*_i_ values for these compounds are shown in Figure 2. The most potent compound is 5-hydroxy-NQ with a *K*_i_ value of 1.8 µM [19]. Anthraflavic acid (2,6-dihydroxyanthracene-9,10-dione, Figure 2), a naturally occurring plant phenol, inhibited aryl hydrocarbon hydroxylase (AHH) activity in microsomes prepared from control and 3-methylcholanthrene-pretreated SENCAR mice with a *K*_i_ value of 165 µM, and inhibited ethoxycoumarin O-deethylase and EROD activities [20].

Fourteen commercially available naphthoquinone and anthraquinone derivatives were evaluated for their inhibition of N-hydroxylation activity of P450 1A1 toward Trp-P-2 (3-amino-1-methyl-5H-pyrido[4,3-b]indole). Among these derivatives, alizarin (IC_50_ = 0.98 µM, Figure 2) and emodin (IC_50_ = 0.96 µM, Figure 2) showed the most effective inhibition of P450 1A1 [21]. An *in vitro* assay also suggested that anthraquinones (especially quinizarin) affected the mutagenicity of IQ mainly through inhibiting the microsomal activating enzyme (P450 1A2) which metabolized IQ to active metabolite *N*-hydroxy-IQ [22].

Using emodin as a lead compound, 12 synthetic naphthoquinone and anthraquinone derivatives were investigated. Compounds 4-amino-1-chloro-3-methylanthraquinone, 4-amino-1-hydroxy-anthraquinone, and 4-((3-methylbutan-2-yl)oxy)-3,4,4a,9a-tetrahydroanthracene-2,9,10(1*H*)-trione were the most potent among the 12 derivatives, with IC_50_ values of 0.40, 2.09, and 0.89 µM for P450 1A1 and 0.53, 0.53, and 1.62 µM for P450 1A2, respectively. 4-Amino-1-chloro-3-methyl-anthraquinone showed mechanism-based inhibition of both P450s 1A1 and 1A2 [23].

Since naphthoquinones and anthraquinones are natural products some of which have been used in Chinese traditional medicine for hundreds of years, their toxicity is much lower than that of PAHs. However, their efficacies for inhibiting P450 family 1 enzymes are also lower than those of PAHs and their structures need to be optimized to obtain highly potent and selective inhibitors of these enzymes.

### 2.3. Stilbenoids

*trans*-Stilbene, (*E*)-1,2-diphenylethene, is another main structural type of non-nitrogen P450 family 1 inhibitors. The first studied stilbenoid as a P450 inhibitor was resveratrol (3,5,4'-trihydroxystilbene), which is a natural product found in red grapes. Resveratrol showed chemopreventive effects in both cardiovascular disease and cancer [24]. Resveratrol exhibited mild inhibition of human P450 1A1 in a dose-dependent manner, with an IC_50_ value of 23 µM for EROD and an IC_50_ value of 11 µM for methoxyresorufin *O*-demethylation (MROD). However, the inhibition of human P450 1A2 by resveratrol was over 50-fold weaker than that of P450 1A1 (IC_50_ 1.2 mM for EROD and 580 µM for MROD) [24]. Mechanism investigation showed that resveratrol inhibited human P450 1A1 activity in a mixed-type inhibition (competitive-noncompetitive) with *K*_i_ values of 9 µM (for competitive inhibition) and 89 µM (for non-competitive inhibition). Resveratrol is also a noncompetitive inhibitor of human P450 1B1 (with *K*_i_ = 23 µM) [24] and a mechanism-based inactivator of cytochrome P450 3A4 (*K*_I_ and *k*_inact_ values of 20 µM and 0.20 min^−1^, respectively) [25]. On the other hand, resveratrol was shown to be an arylhydrocarbon receptor (AhR) antagonist, inhibiting the induction of P450 1A1 [26]. Resveratrol inhibited TCDD-induced *CYP1A1* expression in HepG_2_ cells (*in vitro*) through blocking the induction of DRE (dioxin-responsive element)-driven transcription by AhR [27]. In an animal model, resveratrol was able to abrogate B[a]P-induced P450 1A1 activity and subsequent formation of B[a]P-DNA adducts [28].

**Figure 3:**
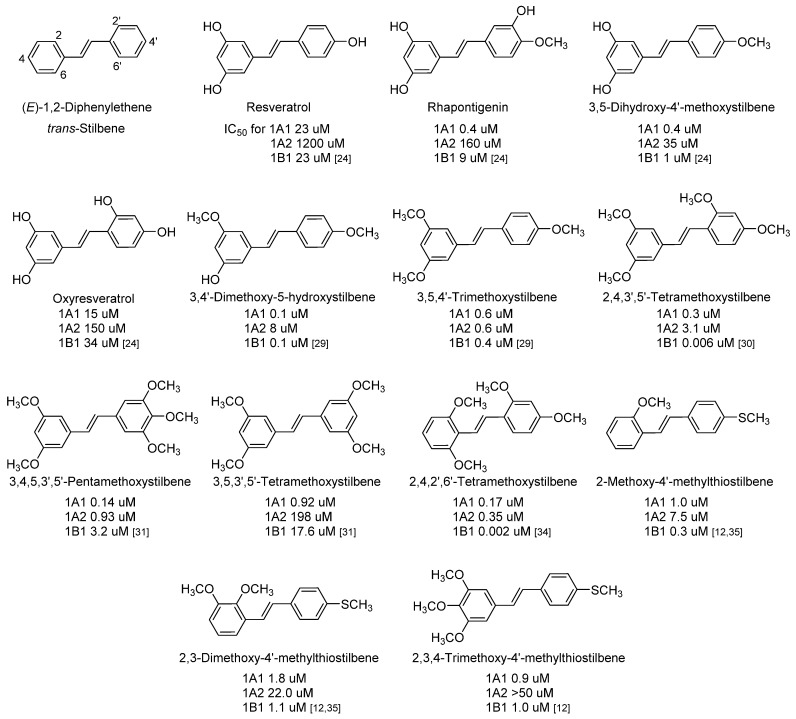
Stilbenoids as P450 1A1, 1A2, and 1B1 inhibitors.

To find more effective inhibitors toward P450 1A1, a group of natural and synthetic hydroxylstilbenes (Figure 3) were investigated for their P450 inhibition activities by Chun *et al*. Monomethylation of the 4'-hydroxyl group (3,5,3'-trihydroxy-4'-methoxystilbene (rhapontigenin) and 3,5-dihydroxy-4'-methoxystilbene) increased the inhibitory activities and kept the selectivity for P450 1A1, while multi-methylation (3,4'-dimethoxy-5-hydroxystilbene and 3,5,4'-trimethoxystilbene) improved the inhibitory potency toward both P450s 1A1 and 1A2, resulting in the loss of selectivity toward P450 1A1. The inhibitory activities toward P450 1B1 were also determined in this study [29]. Among these stilbenes, rhapontigenin extracted from *Rheum undulatum* was shown to be a mechanism-based inactivator of P450 1A1 with an IC_50_ value of 0.4 μM [30]. Rhapontigenin showed 400-fold selectivity toward P450 1A1 over P450 1A2 (IC_50_ = 160 μM) and 23-fold selectivity toward P450 1A1 over P450 1B1 (IC_50_ = 9 μM) [29].

2,4,3',5'-Tetramethoxystilbene (2,4,3',5'-TMS, Figure 3), a methoxy derivative of oxyresveratrol was found to be the most selective competitive inhibitor of P450 1B1, with an IC_50_ value of 6 nM in a group of 3,5-dimethystilbene derivatives [30,31]. 2,4,3',5'-TMS exhibited 50-fold selectivity for P450 1B1 over P450 1A1 (IC_50_ = 300 nM) and 500-fold selectivity for P450 1B1 over P450 1A2 (IC_50_ = 3 μM) in EROD assay [30]. 2,4,3',5'-TMS strongly inhibited 4- and 2-hydroxylation of 17β-estradiol by P450 1B1-expressing membranes or purified P450 1B1 [30]. 2,4,3',5'-TMS also showed suppression of TCDD (2,3,7,8-tetrachlorodibenzodioxin)-induced *CYP1B1* expression in MCF-7 cells and HL-60 cells [32]. In the same study, 3,4,5,3',5'-pentamethoxystilbene (PMS) and 3,5,3',5'-tetramethoxystilbene were found to be selective inhibitors toward P450 1A1 (Figure 3) [31]. PMS, a heavily-studied compound, produced a significant inhibition of EROD activities with IC_50_s of 0.14, 934, and 3.2 μM for P450s 1A1, 1A2, and 1B1, respectively. Moreover, PMS significantly suppressed P450 1A1-mediated EROD activity and *CYP1A1* gene expression induced by TCDD in HepG_2_ cells [33].

2,4,2',6'-Tetramethoxystilbene is another potent and specific inhibitor of P450 1B1. 2,4,2',6'-TMS exhibited potent and selective inhibition of EROD activity of P450 1B1 with an IC_50_ value of 2 nM. 2,4,2',6'-TMS exhibited 175-fold selectivity for P450 1B1 over 1A1 (IC_50_, 350 nM) and 85-fold selectivity for P450 1B1 over 1A2 (IC_50_, 170 nM). 2,4,2',6'-TMS significantly suppressed EROD activity and *CYP1A1* and *CYP1B1* induction by TCDD in human tumor cells such as HepG_2_ and MCF-10A [34].

Some stilbene derivatives with a methylthio substituent were shown to be selective and potent inhibitors of P450 family 1 [35]. Among this series of compounds studied, 2-methoxy-4'-methylthiostilbene, 2,3-dimethoxy-4'-methylthiostilbene, and 2,3,4-trimethyoxy-4'-methylthiostilbene (Figure 3) were the most potent competitive inhibitors of P450 family 1 enzymes [12,35]. Especially, 2,3,4-trimethoxy-4'-methylthiostilbene was the most selective inhibitor of P450s 1A1 and 1B1, displaying extremely low affinity toward P450 1A2 [12].

In summary, 2,4,3',5'-tetramethoxystilbene and 2,4,2',6'-tetramethoxystilbene appear to be potent and specific inhibitors of P450 1B1 [30,34]. Rhapontigenin and 2,3,4-trimethyoxy-4'-methyl-thiostilbene are the most selective inhibitors toward P450s 1A1 and 1B1 over P450 1A2 [29,34]. Meanwhile, 3,5,4'-trimethoxystilbene exhibits comparable inhibitory activities toward all of the family 1 P450 enzymes. Because of the excellent selectivity toward P450s 1A1 or/and 1B1 and the capability of inhibiting AhR-induced Phase I metabolizing enzyme expression, *trans*-stilbenes are considered to be promising potential chemopreventive agents for both environmental carcinogen-induced and (17β-estradiol) E2-related tumorigenesis.

### 2.4. Flavonoids

Flavonoids are widely present in fruits, nuts, vegetables, and medical herbs. These compounds have been described as health-promoting as well as disease-preventing dietary supplements [36]. The relative safety of flavonoids has allowed them to be used as therapeutic agents in traditional and modern medicine. Evidence indicates that certain flavonoids have high potency and selectivity for inhibition of P450 1A enzymes. This fact may have important implications for cancer prevention, as well as other pharmacological and toxicological effects [37].

**Figure 4:**
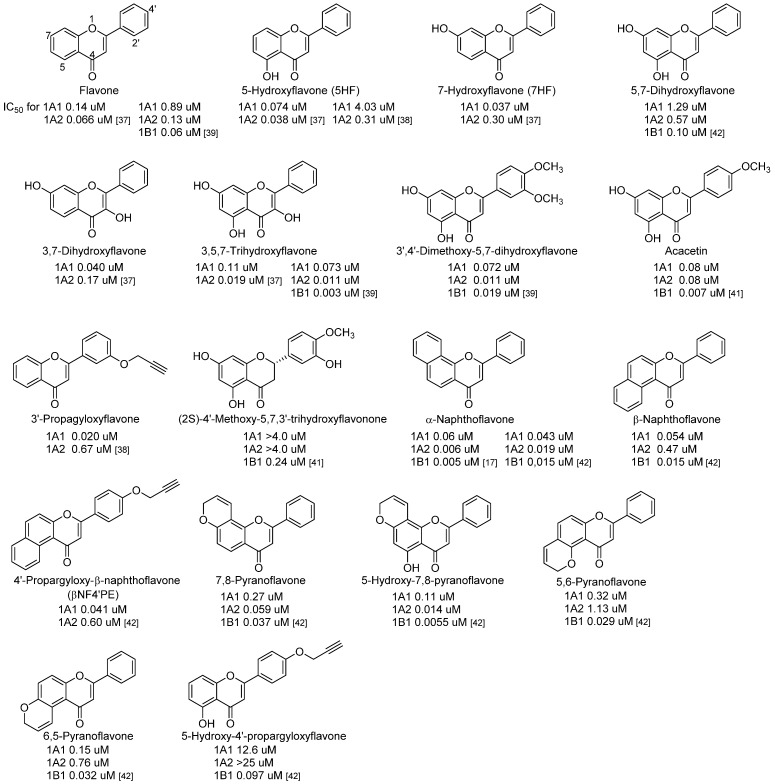
Structures of flavonoid inhibitors.

By contrast with stilbene, the flavone core is a less potent inhibitor of P450 1A1 (IC_50_ = 0.14 µM) than P450 1A2 (IC_50_ = 0.066 µM). Hydroxyflavones were investigated as well, however they showed diverse selectivity toward P450 1A subfamily members. For example, 5-hydroxyflavone and 5,7-dihydroxyflavone showed higher inhibition activity and selectivity toward P450 1A2 compared to P450 1A1 [37,38,39]. Whereas, 7-hydroxyflavone and 3,7-dihydroxyflavone exhibited 4–8 fold greater selectivity in their inhibition of P450 1A1 over 1A2 [37,38]. 3,5,7-Trihydroxyflavone (galangin, Figure 4) showed the highest potency toward P450 1A2. The inhibition of the MROD activity of P450 1A2 by galangin was mixed-type, with a *K*_i_ value of 0.008 µM. Galangin showed a 5-fold selectivity in its inhibition of P450 1A2 over 1A1 [37,39]. 2'-, 3'-, and 4'-methyoxyflavones exhibited similar inhibitory activities toward P450s 1A1, 1A2, and 1B1 as the flavones core, while the introduction of a 4'-methoxy- or 3',4'-dimethoxy group(s) into 5,7-dihydroxyflavone yielded active inhibitors of P450 1B1 with IC_50_ values of 0.014 and 0.019 µM, respectively [39]. Six flavonyl propargyl ethers were also investigated for their inhibitory activities toward P450s 1A1 and 1A2. 3'-Propargyloxyfavone (3'-PF) was the most potent inhibitor among these compounds. 3'-PF exhibited a 33-fold greater selectivity in its inhibition of P450 1A1 over P450 1A2, and it inhibited P450 1A1 in a mechanism-based manner [38].

An intergroup comparative study was carried out on the inhibition of P450 1B1 by flavonoid, stilbenoid, pyrene, naphthalene, phenanthrene, and biphenyl derivatives [40]. The results showed that 3,5,7-trihydroxyflavone, 4'-methoxy-5,7-dihydroxyflavone, and 3',4'-dimethoxy-5,7-dihydroxyflavone were more active than flavone in interacting with P450 1B1. In a study of six naturally occurring flavonoids (acacetin, diosmetin, eriodictyol, hesperetin, homoeriodictyol, and naringenin), flavonone homoeriodictyol selectively inhibited P450 1B1 with a relatively high IC_50_ of 0.24 µM [41].

Several naphthoflavone compounds acting as inhibitors of P450 family 1 enzymes have been investigated. α-Naphthoflavone exhibited a 10-fold higher inhibition of P450 1A2 than P450 1A1. However, α-naphthoflavone did not show selectivity for inhibiting P450 1A2 compared with P450 1B1 [17,42]. On the contrary, β-naphthoflavone exhibited higher inhibition of P450 1A1 than 1A2 [42]. To clarify the relationship between structural characteristic and selectivity, a series of pyranoflavone and 5-hydroxypyranoflavone derivatives were synthesized and examined for inhibition of P450s 1A1, 1A2, and 1B1. The results showed that α-naphthoflavone-like and 5-hydroxyflavone derivatives preferentially inhibited P450 1A2, while β-naphthoflavone-like flavone derivatives selectively inhibited P450 1A1. Interestingly, a highly selective P450 1B1 inhibitor, 5-hydroxy-4'-propargyloxyflavone (5H4'FPE), was identified [42]. A study of four naphthoflavone propargyl ethers showed that 4'-propargyloxy-β-naphthoflavone (βNF4'PE, Figure 4) exhibited high inhibitory activity and selectivity toward P450 1A1 [39]. In comparison to stilbenes, flavonoids possess more affinity toward P450 1A2 compared to P450 1A1. Thus modifying flavone derivatives seems to be a logical approach to the design of selective P450 1A2 inhibitors as well as to study this enzyme’s active site cavity.

As well-documented P450 inhibitors, the effects of flavonoids on AhR function and P450 family 1 enzyme expression were also investigated in many studies [36]. Ciolino *et al*. reported that galangin (3,5,7-trihydroxyflavone) caused an increase in the amount of *CYP1A1* mRNA, but inhibited the induction of *CYP1A1* mRNA by DMBA or by TCDD in MCF-7 human breast cancer cells. Galangin also inhibited the DMBA- or TCDD-induced transcription of a reporter vector containing the *CYP1A1* promoter [43]. Quercetin and kaempferol are two of most abundant dietary flavonoids. Quercetin caused a time- and concentration-dependent increase in the level of *CYP1A1* mRNA and P450 1A1 enzyme activity in MCF-7 cells. However, kaempferol inhibited the TCDD-induced *CYP1A1* transcription instead of affecting normal *CYP1A1* expression [44]. Baicalein (5,6,7-trihydroxyflavone, isolated from the plant *Scutellariae baicalensis*) reduce the *CYP1A1/1B1* mRNA expression induced by DMBA, and the mRNA abundance for *CYP1A1* appeared to be more responsive than that of *CYP1B1* [45]. A flavone derivative, aminoflavone (Figure 4), also caused induction of *CYP1A1* and *CYP1B1* transcription through the AhR pathway [46]. Because of the two-way action of flavonoids on P450 enzyme inhibition and *CYP* gene expression, the true effects of a certain flavonoid in cell or *in vivo* are complex and need further investigation.

### 2.5. Coumarins

**Figure 5:**
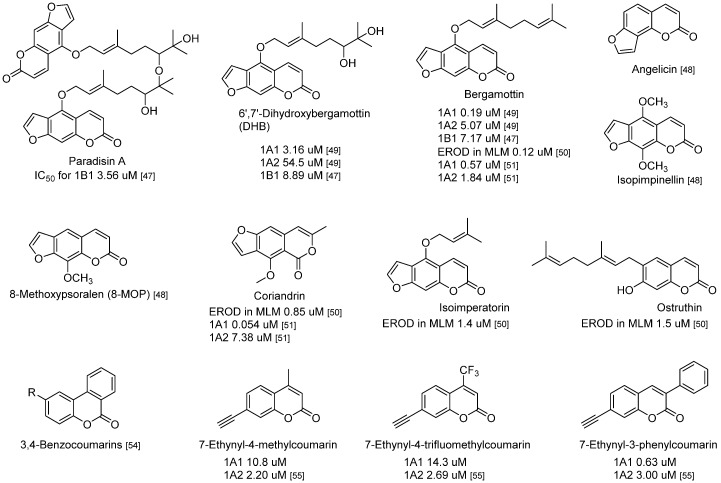
Coumarin derivatives as P450 family 1 inhibitors (MLM, mice liver microsome).

The most investigated coumarins, furocoumarin derivatives, were isolated from grapefruit juice and showed the capacity to inhibit the activity of certain human cytochrome P450 enzymes including P450 family 1 enzymes [47]. Among these compounds, paradisin A, 6',7'-dihydroxybergamottin (DHB), and bergamottin (Figure 5) showed considerable inhibition of P450 1B1-mediated EROD activity with IC_50_ values of 3.56 µM, 8.89 µM and 7.17 µM, respectively [47]. It has been reported that furocoumarins, angelicin, bergamottin, isopimpinellin, and 8-methoxypsoralen (8-MOP), effectively inhibited the catalytic activity of P450 1A1, but induced *CYP1A1* gene expression via the AhR or non-AhR pathway [48]. As a recent study described, bergamottin and DHB inhibited alkoxyresorufin *O*-dealkylase activity of P450s 1A1 and 1A2 as well as the mutagenic effect of 3-MC and B[a]P in the Ames Test [49]. Cai *et al*. reported that several naturally occurring coumarins found in the human diet, including coriandrin, bergamottin, isoimperatorin, and ostruthin (Figure 5), were effective inhibitors of hepatic EROD activity *in vitro* in hepatic tissues from SENCAR mice. Notably, bergamottin (IC_50_ = 0.12 µM) and coriandrin (IC_50_ = 0.85 µM) were approximately as potent as α-naphthoflavone (IC_50_ = 0.25 µM) [50]. Coriandrin was found to selectively inhibit P450 1A1-mediated EROD activity and to be a mechanism-based inactivator of this enzyme *in vitro* [51]. Contrary to flavonoids, coumarins are more efficient inhibitors of P450s 1A1 and 1B1 compared to P450 1A2.

The effect of furocoumarins on P450 expression was compared to flavones. Bergamottin and coriandrin were found to inhibit metabolism of B[a]P and DNA adduct formation in cultured mouse epidermal keratinocytes [52]. Bergamottin inhibited DMBA metabolism and blocked DNA adduct formation in Hepa-1 cells, but had little effect in 10T1/2 cells. In contrast, α-naphthoflavone completely blocked DMBA metabolism and DNA adduct formation in 10T1/2 cells, but had little effect in Hepa-1 cells [53]. However, imperatorin and isopimpinellin (Figure 5) inhibited DMBA bioactivation in both cell lines [53].

The investigated synthetic coumarins included 6-substituted 3,4-benzocoumarins [54] and 7-ethynylcoumarins [55]. A group of ethynyl coumarin derivatives was found to effectively inhibit P450 1A enzymes. Specifically, 7-ethynyl-4-trifluomethylcoumarin and 7-ethynyl-4-methylcoumarin exhibited higher inhibition of P450 1A2 over 1A1, while 7-ethynyl-3-phenylcoumarin showed higher potency of inhibition for P450 1A1 over 1A2 [55].

### 2.6. Alkaloids

Alkaloids are a large group of naturally occurring *N*-containing compounds. Since some of these compounds possess planar structural characteristics, they have the potential to be P450 inhibitors [16]. Ellipticine, a pyridocarbazole, was the first identified alkaloid inhibitor of P450 family 1 enzymes. It was isolated from *Ochrosia elliptica Labill*, well-known as an antineoplastic agent [56]. Although ellipticine showed a strong inhibition of EROD activity in rat liver microsomes (IC_50_ = 0.11 µM), its inhibitory potential was non-specific and P450s from subfamilies 2B, 2D, and 3A were also inhibited by this compound [57].

A caffeine analog, furafylline, was the first selective P450 1A2 inhibitor identified. The first indication that it might inhibit P450 1A2 *in vivo* was that furafylline dramatically increased the half-life (7- to 10-fold) of caffeine in healthy volunteers [58]. Furafylline is a potent inhibitor of P450 1A2 phenacetin O-deethylase activity (IC_50_ = 0.07 µM) in human liver microsomes. However, P450s 1A1, 2A6, 2C8, 2D6, and 3A3/4 were not inhibited by furafylline (IC_50_ > 500 µM) [59]. In another study, furafylline was found to be a mechanism-based inhibitor of P450 1A2 [60]. The inactivation of P450 1A2 by this compound was characterized by a *K*_i_ of 23 µM and a *k*_inact_ of 0.87 min^−1^.

The indole alkaloid melatonin (*N*-acetyl-5-methoxytryptamine) is an endogen, which is a secretory product of the pineal gland [60]. Melatonin inhibited P450 family 1 enzymes, with apparent *K*_i_ values of 59 (1A1), 12 (1A2), and 14 µM (1B1). Treatment of MCF-10A human mammary epithelial cells with 300 μM of melatonin did not affect basal or B[a]P-inducible *CYP1A1* or *CYP1B1* expression [61].

**Figure 6:**
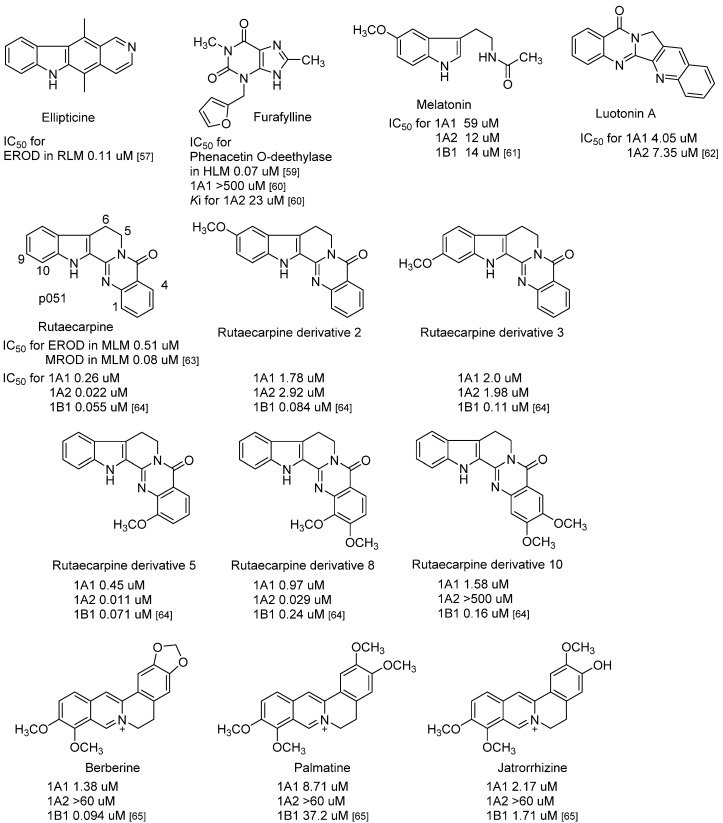
Structures of alkaloids as P450 family 1 inhibitors (RLM, rat liver microsome; HLM, human liver microsome; MLM, mouse liver microsome).

Luotonin A, a pyrroloquinolinequinoline alkaloid originally isolated from *Peganum nigellastrum*, is a natural inhibitor of DNA topoisomerase I. Luotonin A inhibited P450 1A2-catalyzed phenacetin O-deethylation with an IC_50_ of 6.3 μM in human liver microsomes, but did not inhibit it in a time-dependent manner. Luotonin A was found to decrease human recombinant cDNA-expressed P450 1A1- and 1A2-catalyzed phenacetin O-deethylase activities simultaneously, with IC_50_ values of 4.05 and 7.35 μM, respectively (Figure 6) [62].

Of the alkaloids, quinazolinocarboline rutaecarpine (a COX-2 inhibitor) showed the most potent and selective inhibitory effects on P450-catalyzed MROD and EROD activities in untreated mouse liver microsomes. The IC_50_ ratio of MROD (IC_50_ = 0.08 µM) to EROD (IC_50_ = 0.51) was more than 6 [63]. A series of synthetic rutaecarpines were evaluated for inhibition of P450s 1A1, 1A2, and 1B1. Among the derivatives, 10- and 11-methoxyrutaecarpine (Figure 6) were the most selective P450 1B1 inhibitors. 1-Methoxyrutaecarpine and 1,2-dimethoxyrutaecarpine were the most selective P450 1A2 inhibitors [64].

Berberine, palmatine, and jatrorrhizine are antimicrobial protoberberine alkaloids, present in several medicinal herbs, such as *Coptis chinensis* (Huang-Lian) and *Hydrastis canadensis* (goldenseal). These protoberberines inhibited P450 1A1- and 1B1-catalyzed EROD activities, whereas CYP1A2 activity was barely affected. Kinetic analysis revealed that berberine noncompetitively inhibited EROD activities of P450s 1A1 and 1B1, whereas palmatine and jatrorrhizine caused either competitive or mixed-type inhibition. Among protoberberines, berberine caused the most potent and selective inhibitory effect on P450 1B1 with the lowest *K*_i_ value of 44 nM [65]. *In vivo* assays showed that AhR was activated by high doses of berberine (10–50 µM) after 6 and 24 h of incubation as revealed by *CYP1A1* mRNA expression (HepG_2_ cells) and AhR-dependent luciferase activity (H4IIE.luc cells). Whereas, berberine was a potent inhibitor (IC_50_ = 2.5 µM) of P450 1A1 catalytic activity (EROD) in HepG_2_ cell culture and in recombinant P450 1A1 protein [66].

### 2.7. Other Natural Products

**Figure 7:**
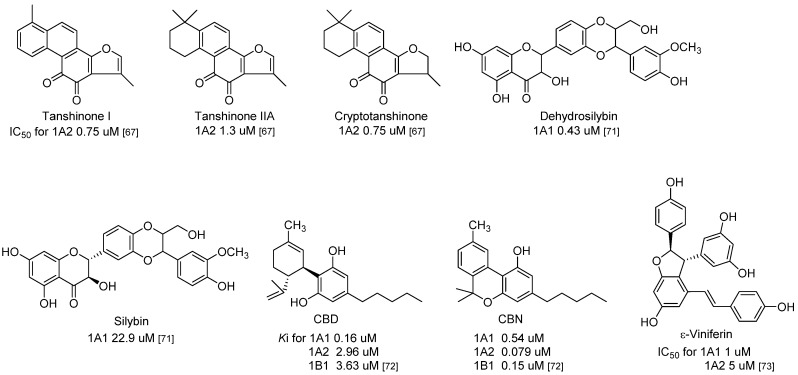
Other naturally occurring products as P450 family 1 inhibitors.

Ueng *et al*. isolated and identified a group of P450 inhibitors from danshen (the dried root of *Salvia miltiorrhiza*), which is used in the treatment of angina pectoris, hyperlipidemia, and acute ischemic stroke. Among the seven components of danshen extract, tanshinone I, tanshinone IIA, and cryptotanshinone (Figure 7) were shown to be potent competitive inhibitors of P450 1A2 (IC_50_ = 0.75, 1.3, and 0.75 µM, respectively) [67]. In *Escherichia coli* membranes expressing bicistronic human P450 1A enzymes, tanshinone IIA inhibited EROD activity of P450 1A1 with an IC_50_ 48 times higher than that for P450 1A2 [68]. *In vitro* assays showed that tanshinones caused a significant time- and concentration-dependent increase in the amount of *CYP1A1* and *CYP1A2* expression in HepG_2_ cells through a transcriptional activation mechanism [69]. Oral treatment of tanshinone IIA caused a dose-dependent increase of liver microsomal MROD activity in the arylhydrocarbon (Ah)-responsive C57BL/6J (B6) mice but not in (Ah)-nonresponsive DBA/2J (D2) mice. These results demonstrated that induction of P450 1A2 by tanshinone IIA depended on the Ah-responsiveness and occurred at pre-translational level [70].

The compounds silybin and dehydrosylibin purified from the extract of the fruit of *Silybum marianum* showed considerable inhibition of human recombinant protein P450 1A1, with IC_50_ values of 22.9 µM and 0.43 µM, respectively. Silybin and dehydrosylibin inhibited basal and dioxin-inducible P450 1A1 catalytic activity in human keratinocytes (HaCaT) and human hepatoma cells (HepG_2_). The inhibitory effects of tested compounds were more pronounced in HaCaT cells than in HepG_2_ cells, and dehydrosilybin was a much stronger inhibitor than silybin [71].

Inhibitory effects of cannabidiol (CBD) and cannabinol (CBN), the two major constituents in marijuana, on catalytic activities of human cytochrome P450 family 1 enzymes were investigated. These cannabinoids inhibited EROD activity of recombinant P450s 1A1, 1A2, and 1B1 in a competitive manner. CBD most potently inhibited P450 1A1; the apparent *K*_i_ value (0.16 µM) was at least one-seventeenth of the values for the other P450 1 enzymes. On the other hand, CBN decreased the activity of P450s 1A2 and 1B1 (*K*_i_ = 0.079 and 0.15 µM, respectively) more effectively than P450 1A1 (*K*_i_ = 0.54 µM) [72]. ε-Viniferin, a dimer of resveratrol, has also been studies and showed higher inhibitory activities toward P450s 1A1 and 1A2 than resveratrol [73].

### 2.8. Drugs

Because of potential drug-drug interactions, the effects of drugs on P450 enzymes are of great importance in pre-clinical pharmacological studies as well as clinical trials. Since P450 1A2 is one of the major drug metabolizing enzymes, it has been the most investigated enzyme for the inhibitory effect of drugs.

**Figure 8:**
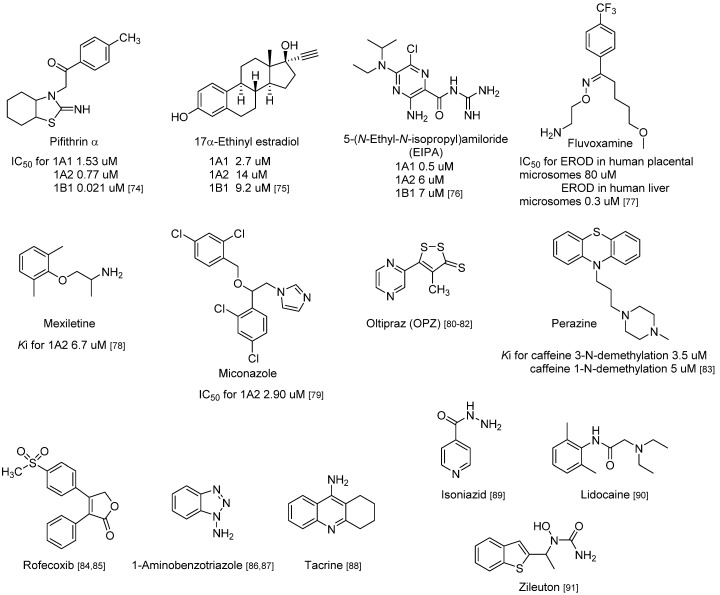
Drugs as P450 family 1 inhibitors.

Pifithrin α (PFTα, Figure 8), a p53 inhibitor, decreased P450s 1A1, 1A2 and 1B1 catalytic activities in a concentration-dependent manner. PFTα showed a selective inhibitory effect on P450 1B1 activity (IC_50_ = 0.021 µM), compared with the activities of P450s 1A1 (IC_50_ = 1.53 µM) and 1A2 (IC_50_ = 0.77 µM) [74]. The contraceptive agent 17α-ethinyl estradiol (EE, Figure 8) appeared to cause selective inhibition of P450 1A1 (IC_50_ = 2.7 µM) over P450 1A2 (IC_50_ = 14 µM) [75]. The amiloride (a diuretic drug) derivative EIPA (5-(*N*-ethyl-*N*-isopropyl)amiloride, Figure 8) was found to cause a potent and dose-dependent inhibition of P450 1-related EROD activity in both liver cells and microsomes [76]. The antidepressant fluvoxamine (Figure 8) was a potent inhibitor of P450 1A2-mediated EROD activity (IC_50_ = 0.3 μM) in human liver microsomes, but caused weak inhibition of P450 1A1-mediated EROD activity in human placental microsomes [77]. Anti-arrhythmic agent mexiletine (Figure 8) preferentially inhibited P450 1A enzymes in hepatic microsomes. The P450 1A2-mediated MROD activity was inhibited by mexiletine with a *K*_i_ of 6.7 μM [78]. The antifungal drug miconazole (Figure 8) appeared to be a competitive inhibitor against P450 1A2-mediated EROD and P450 2D6-mediated debrisoquine 4-hydroxylation with IC_50_ values of 2.90 and 6.46 µM, respectively [79].

The chemoprotective agent oltipraz (OPZ, Figure 8) was a potent inhibitor of xenobiotic and procarcinogen oxidation activities in recombinant human P450 enzymes, with P450 1A2 being more affected than P450s 1A1, 1B1, 2E1, or 3A4 [80]. The success of OPZ as a chemoprotective agent against the induction of hepatocarcinogenesis by aflatoxin B_1_ (AFB_1_) in rats depended principally on its ability to enhance detoxification by inducing phase II enzymes, especially glutathione transferases. However, OPZ also had an important inhibitory effect on the major P450 enzymes responsive for AFB_1_ metabolism, in primary cultures of human hepatocytes [81,82].

The phenothiazine neuroleptic perazine (Figure 8) showed potent inhibition of caffeine 3-*N*-demethylation (*K*_i_ = 3.5 μM) and caffeine 1-*N*-demethylation (*K*_i_ = 5 μM) in both human liver microsomes and cDNA-expressed P450 1A2 (supersomes) [83]. Other investigated drugs (Figure 8) included rofecoxib [84,85], 1-aminobenzotriazole (ABT) [86,87], tacrine [88], isoniazid [89], lidocaine [90], and zileuton [91], *etc*. Since a large number of drugs are metabolized by P450 1A2, concerns have been raised about the potential interactions between these inhibitors and other P450 1A2 substrate drugs.

### 2.9. Other Synthetic Compounds

DMPVT (2-[2-(3,5-dimethoxyphenyl)vinyl]thiophene), an analog of stilbene, significantly inhibited EROD activities with IC_50_ values of 61, 11, and 2 nM for P450s 1A1, 1A2, and 1B1, respectively. The DMBA-induced EROD activity in rat lung microsomes was also significantly inhibited by DMPVT in a dose-dependent manner. The mechanism of inhibition by DMPVT were non-competitive for the three enzymes [92].

**Figure 9:**
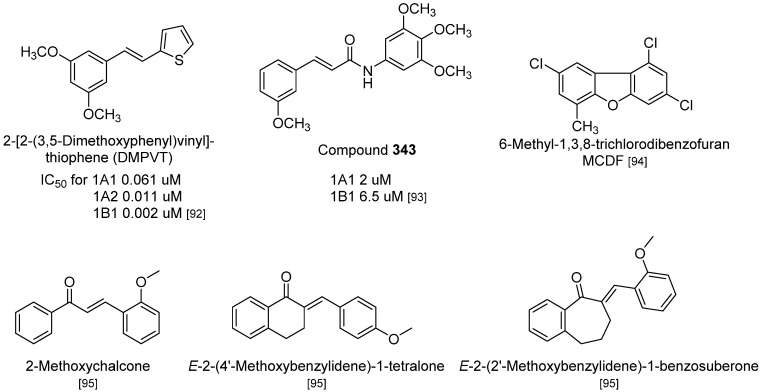
Other synthetic inhibitors.

The compound 343 (Figure 9) described by Sienkiewicz *et al*. caused a concentration-dependent decrease in the EROD activity of P450 1A1 (IC_50_ = 2 µM) and 1B1 (IC_50_ = 6.5 µM). There was only a modest inhibition of P450 1A2 activity [93]. Compound 343 also inhibited the upregulation of cytochrome P450 enzyme activities and *CYP* mRNA levels in cells treated with the AhR ligands, DMBA or TCDD [93].

Some chalcone, tetralone, and benzosuberone derivatives as well as 6-methyl-1,3,8-trichloro-dibenzofuran (MCDF) exhibited inhibition of metabolic activation of carcinogen DMBA by P450 family 1 enzymes and TCDD-induced *CYP1A* expression, respectively. [94,95].

### 2.10. Mechanism-Based Inhibitors (MBIs)

As hemoprotein monooxygenases, cytochrome P450s are responsible for the oxidation of a wide variety of xenobiotics and endogenous compounds. Once the oxidation reaction with a potential inhibitor produces reactive intermediate(s) that can covalently bond to the P450 active site, irreversible inactivation occurs [96,97]. This is the basis of mechanism-based inactivation, and these potential inhibitors are known as mechanism-based or suicide inhibitors. Because of the complete irreversible inactivation of the P450 enzyme, a mechanism-based inhibitor is much stronger than a competitive inhibitor with the same affinity for the enzyme. Thus, mechanism-based inhibitors are used in the design of specific enzyme-targeted drugs since they only affect the enzyme which can metabolize them into oxygenated active intermediate(s). Mechanism-based inhibitors are also of great interest to researchers because of their utility in elucidating the characteristics of enzyme active site cavities and enzymatic reaction mechanisms.

**Figure 10:**
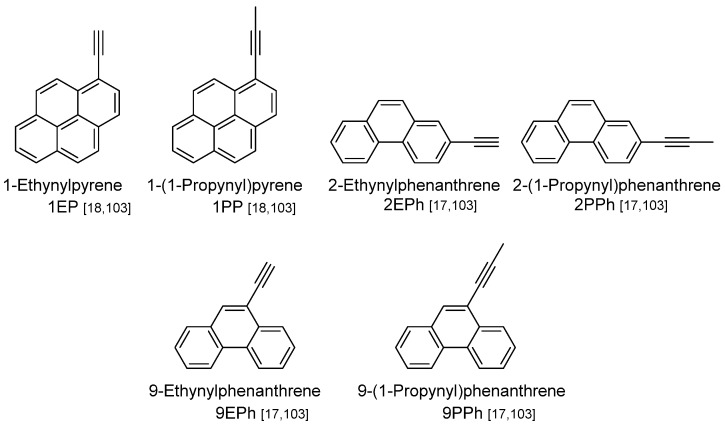
Selective MBIs with acetylenic group.

Since mechanism-based irreversible inactivation (suicide inhibition) is characterized by a pseudo-first-order time-dependent and NADPH-dependent loss of the enzymatic activity, a large number of MBIs (Figure 10) have been identified according to these criteria. Some of these compounds have been reviewed in the earlier sections of this review. These MBIs include anthraquinone derivatives (4-amino-1-chloro-3-methylanthraquinone [23]), stilbenoids (rhapontigenin [29]), flavones (7-hydroxyflavone, 3-flavon propargyl ether [38]), coumarins (coriandrin [51]), alkaloids (furafylline [60]), synthetic drugs (1-aminobenzotriazole [86], tacrine [88], and oltipraz [80]).

Many mechanism-based inhibitors were intentionally designed and synthesized employing a basic reactive unit, acetylene. The triple bond of these terminal acetylenes is oxidized by the P450 enzyme to form a reactive ketene intermediate by a 1,2-hydrogen shift. The ketene intermediate can then react with the heme nitrogen resulting in a time-dependent destruction of the heme [98,99,100] or covalently bond with a nucleophilic residue of the active site protein, thereby inactivating the enzyme [101,102]. The other important component of these compounds is a planar conjugated system, fitting the narrow active site cavity of P450 family 1 enzymes. A large group of ethynyl and 1-propynyl pyrenes, phenanthrenes, naphthalenes, and biphenyls were investigated in a series of studies on P450s 1A1, 1A2, and 1B1 [17,18,103,104]. The results showed that inhibition mechanism varied for the different compounds and different enzymes. For example, 1EP (1-ethynylpyrene) is a mechanism-based inhibitor of P450 1A1, but a competitive inhibitor of P450s 1A2 and 1B1. While 1PP (1-(1-propynyl)pyrene) is a mechanism-based inhibitor of P450s 1A1 and 1A2, but a competitive inhibitor of P450 1B1 [18,103]. It was generally observed that replacing the terminal hydrogen of aryl acetylenes with a methyl group to convert ethynes into propynes enhanced the inhibition of P450 1A enzymes; in some instances, this modification converted a reversible inhibitor into a suicide inhibitor [103]. It was also observed that 2EPh (2-ethynylphenanthrene) and 2PPh (2-(1-propynyl)phenanthrene) more potently inhibited P450 1A1 than 1A2, whereas 9EPh (9-ethynylphenanthrene) and 9PPh (9-(1-propynyl)phenanthrene) selectively inhibited P450 1A2 over 1A1 [17,103]. This suggests that the molecular shape of an inhibitor is critical for the inhibitor’s selectivity towards P450s 1A1 and 1A2, which will be further discussed in the following section. The studies on the 7-ethynylcoumarins and flavonyl propargyl ethers also provided mechanism-based inhibitors of P450 1As, and certain structural characteristics of P450 1A1 and 1A2’s active site cavities [38,55].

## 3. Structural Characteristics of P450s 1A1 and 1A2 Inhibitors

The crystal structures of P450s 1A1, 1A2, and 1B1 have been elucidated in the recent years, and the apparent active site sizes ranked as 1A1 (524 Å^3^) > 1B1 (469 Å^3^) > 1A2 (441 Å^3^) [1,105,106]. These crystal structures are very useful for exploring active sites, enzyme-ligand binding, and related interactions. P450s 1A1 and 1A2 are most similar, sharing 72% amino acid sequence identity, while P450 1B1 has relatively low amino acid sequence identity with both P450s 1A1 (38%) and 1A2 (37%) [1]. However, in terms of the 3D structures and the function of enzymes, P450 1A1 shows much more similarity to P450 1B1 than P450 1A2. In fact, P450 1A2 is largely responsible for the metabolism of aromatic amines, whereas P450s 1A1 and 1B1 metabolize polycyclic aromatic hydrocarbons and polyhalogenated aromatic hydrocarbons [2,107,108,109]. Furthermore, observations of crystal structures also show that similarity of active site cavities of P450s 1A1 and 1B1 is considerably more than that of P450s 1A1 and 1A2, indicating that selectively inhibiting P450s 1A1 and 1B1 is much more difficult than P450s 1A1 and 1A2 [1]. On the other hand, the P450 1A1 and 1A2 encoding genes (*CYP1A1* and *CYP1A2*) are located head-to-head on human chromosome 15q24.1, suggesting a close correlation of *CYP1A1* and *CYP1A2* expression, while *CYP1B1* gene is located on human chromosome 2p22.2. Thus, a certain gene expression regulator, such as an AhR ligand, that leads to the same effects on *CYP1A1* and *1A2* transcription, probably leads to less or reverse effect on transcription of *CYP1B1*. Since some P450 inhibitors also regulate *CYP* gene expression, the effects of a single chemical entity on the level and activity of a P450 enzyme are complex and hard to predict.

Herein, we attempted to explore the inhibitors’ selectivity toward P450s 1A1 and 1A2 by a meta-analysis. Due to the variations of the inhibitory activity data, originated from the use of different experimental methods in different research groups, the absolute IC_50_ values are not usable for this analysis. Thus, we defined the selectivity ratio (the ratio of IC_50_ values for P450s 1A1 and 1A2) in the same study as a parameter. The selective inhibitors were classified into two groups, IC_50(1A1)_/IC_50(1A2)_ < 0.2 as selective 1A1 inhibitors (Group 1), and IC_50(1A1)_/IC_50(1A2)_ > 5 as selective 1A2 inhibitors (Group 2), respectively. An alignment was carried out in each group, and the aligned images exhibited the structural characteristics of each group, which showed the features of the active site cavities of P450s 1A1 and 1A2. Twenty-seven compounds were selected as potential 1A1 inhibitor group, and 23 compounds were selected as potential 1A2 inhibitor group. Group 1 included berberine, palmatine, jatrorrhizine, rutaecarpine derivative 10, coriandrin, 7-ethynyl-3-phenylcoumarin, 17α-ethinyl estradiol, EIPA, 7-hydroxyflavone, isorhamnetin, 3'-propagyloxyflavone, β-naphtho- flavone, βNF4'PE, 6,5-pyranoflavone, 2PPh, CBD, resveratrol, rhapontigenin, 3,5-dihydroxy-4’-methoxystilbene, oxyresveratrol, 3,4'-dimethoxy-5-hydroxystilbene, 2,4,3',5'-tetramethoxystilbene, 3,4,5,3',5'-pentamethoxystilbene, 3,5,3',5'-tetramethoxystilbene, 2-methoxy-4'-methylthiostilbene, 2,3-dimethoxy-4'-methylthiostilbene, and 2,3,4-trimethoxy-4'-methylthiostilbene. Group 2 included pyrene, 4PP, phenanthrene, 2EPh, 2PPh, benz[a]anthracene, furafylline, rutaecarpine, rutaecarpine derivative 5, rutaecarpine derivative 8, 7-ethynyl-4-methylcoumarin, 7-ethynyl-4-trifluomethyl-coumarin, melatonin, fluvoxamine, flavone, 5-hydroxyflavone, 3,5,7-trihydroxyflavone, tanshinone IIA, α-naphthoflavone, 7,8-pyranoflavone, 5-hydroxy-7,8-pyranoflavone, CBN, and DMPVT.

**Figure 11:**
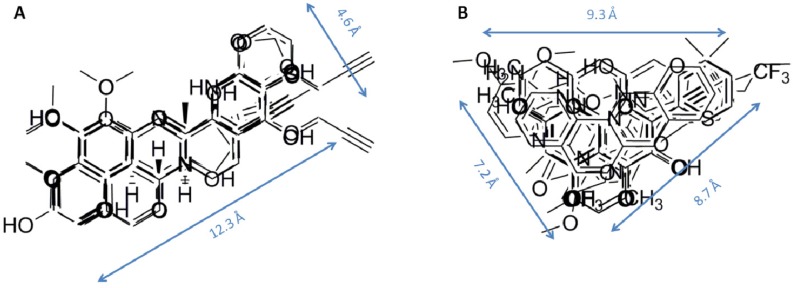
Alignment images of group 1 and group 2 inhibitors. (**A**) Group 1 represents selective P450 1A1 inhibitors; (**B**) Group 2 represents selective P450 1A2 inhibitors.

The inhibitor structures were sketched in ChemDraw 13.0 (PerkinElmer, Inc., Waltham, MA, USA), and alignment was performed manually. The aligned images (Figure 11) obviously show that the skeleton of selective P450 1A1 inhibitors is a long strip shape with 12.3 Å in length and 4.6 Å in width, reflecting the narrow and long cavity of P450 1A1. While the skeleton of selective P450 1A2 inhibitors is a triangle with side lengths 9.3 Å, 8.7 Å, and 7.2 Å, respectively, suggesting a narrow compact cavity for P450 1A2. These results demonstrate the structural characteristics of P450s 1A1 and 1A2 inhibitors as well as the differences of P450s 1A1 and 1A2’s active site cavities, which could not be determined through the analysis of X-ray crystal structures of the two enzymes.

## 4. Conclusions

In this paper, we have reviewed a large number of P450 family 1 enzyme inhibitors and determined the differences between P450s 1A1 and 1A2’s active site cavities by a meta-analysis, which will benefit the future inhibitor development. Since some P450 enzyme inhibitors are inducers of *CYPs* expression, the evaluation of selective P450 family 1 inhibitors should also consider their effects on the expression of *CYP1s* and other related genes. Thus, an integrated in solution, in cell, and *in vivo* bioassay system should be established to comprehensively evaluate the effects of a P450 inhibitor on the enzymes, on the other drugs, and on the related diseases. In addition, because of the notable relationship between P450 1B1 and carcinogenesis, the development of highly selective P450 1B1 inhibitors should be an important focus of research on the family 1 P450 enzymes. Due to the limited data about the selectivity toward P450 1B1, more comparative studies on P450 family 1 inhibitors are needed.
